# Atmospheric moisture transport anomalies and vegetation response in arid coastal ecosystems: insights from the 2017 coastal El Niño in northern Peru

**DOI:** 10.3389/fpls.2026.1804608

**Published:** 2026-04-07

**Authors:** Chong Zhang, Guohe Huang

**Affiliations:** Faculty of Engineering and Applied Science, University of Regina, Regina, SK, Canada

**Keywords:** atmospheric moisture transport, El Niño, leaf area index, vegetation activity, water vapor transport

## Abstract

**Introduction:**

Atmospheric moisture transport governs oceanic evaporation with terrestrial water availability. However, its role in regulating vegetation activity in arid coastal ecosystems is still poorly understood. In particular, how transient circulation anomalies, such as coastal El Niño events, modify atmospheric moisture conditions and relate to vegetation dynamics at broader spatial scales has received limited attention.

**Methods:**

This study combines an event-based analysis of atmospheric moisture transport during the 2017 coastal El Niño in northern Peru with a global statistical analysis of vegetation-moisture relationships. Low-level moisture transport pathways and moisture properties were examined using backward trajectory analysis. Vegetation activity was represented by leaf area index. Spatial correlations between leaf area index and water vapor transport were evaluated within coastal buffer zones and across global land grid cells.

**Results:**

The 2017 coastal El Niño was associated with a clear reorganization of low-level moisture transport pathways, with four dominant trajectory clusters identified compared to three in 2009. The largest equatorial Pacific pathway accounted for approximately 60% of trajectories in 2017, whereas two southeastern Pacific clusters represented nearly 90% of trajectories under normal conditions. Air parcels reaching the Peruvian coast contained substantial higher moisture content, with specific humidity frequently exceeding 15 g/kg compared to generally below 10 g/kg in 2009. This indicates significantly altered near-surface atmospheric conditions. At the global scale, relationships between leaf area index and water vapor transport varied strongly across regions. Positive associations were mainly observed in moisture-limited regions, particularly across large parts of the Southern Hemisphere and several arid coastal zones. In contrast, negative or weak associations dominated humid tropical regions and much of the Northern Hemisphere.

**Discussion:**

These results indicate that atmospheric moisture transport influences vegetation activity in a region-dependent manner. Its role is more pronounced in arid and moisture-limited environments than in humid regions. By linking an event-scale moisture transport anomaly with global vegetation–moisture relationships, this study provides insight into vegetation sensitivity to circulation-driven moisture variability, particularly in arid coastal ecosystems.

## 1 Introduction

Arid and semi-arid coastal ecosystems rank among the most vulnerable environments worldwide. Vegetation growth and recovery in these regions are tightly constrained by limited water supply and strong atmospheric variability (Reynolds et al., 2007; Maestre et al., 2012). Ecological degradation commonly appears as sparse plant cover, enhanced soil erosion, and weakened ecosystem resilience, rendering restoration efforts highly sensitive to hydroclimatic fluctuations (D’Odorico et al., 2013). Precipitation is often treated as dominant control on vegetation dynamics in water-limited systems (Noy-Meir, 1973; Chaves et al., 2003). However, precipitation alone does not fully characterize the atmospheric water environment experienced by vegetation. Growing evidence indicates that broader atmospheric moisture availability, including the transport and convergence of water vapor, plays an important role in regulating land surface processes and ecosystem functioning (Trenberth et al., 2011; Gimeno et al., 2012). This influence may be particularly relevant in coastal regions, where moisture supplied from adjacent oceans can modify near-surface atmospheric conditions even in the absence of local rainfall (Sodemann et al., 2008).

Atmospheric moisture transport connects oceanic evaporation with terrestrial water availability and forms a fundamental component of the land-atmosphere system (Trenberth et al., 2011). Large-scale moisture fluxes influence not only precipitation occurrence but also near-surface humidity, boundary-layer conditions, and evaporative demand, all of which directly affect plant water stress and photosynthetic activity (Seneviratne et al., 2010; Novick et al., 2016). In dry coastal environments, rainfall is often sporadic and insufficient to sustain long-term vegetation recovery (Noy-Meir, 1973; Reynolds et al., 2007). Under such conditions, transient increases in atmospheric moisture supply can temporarily relax hydrological constraints and create short periods that are favorable for vegetation growth (Trenberth et al., 2011). Understanding how these moisture anomalies propagate from the ocean to land, and whether they are reflected in vegetation responses, is therefore critical for assessing ecosystem vulnerability and restoration potential in degraded coastal landscapes (Sivakumar and Stefanski, 2007; Nichols et al., 2018; Tahsin et al., 2018).

Climate variability and extreme ocean-atmosphere events can strongly modify atmospheric moisture transport pathways (Gimeno et al., 2012). Among these, El Niño-Southern Oscillation (ENSO) events remains a key driver of interannual hydroclimatic variability in the tropics and subtropics regions, primarily through its influence on large-scale circulation and moisture advection (Holton and Dmowska, 1989; McPhaden et al., 2006; Cai et al., 2014; Martinez and Dominguez, 2014). El Niño conditions alter large-scale circulation patterns, weaken trade winds, and enhance evaporation over warm ocean surfaces, leading to substantial reorganization of moisture transport from oceanic source regions toward adjacent land areas (Trenberth et al., 1998). These circulation-induced moisture anomalies can persist over weeks to months and extend beyond regions of active precipitation. While the impacts of El Niño on precipitation extremes and flooding have been widely examined, comparatively few studies have been given to how ENSO-related moisture transport anomalies influence terrestrial ecosystems (Holmgren et al., 2001). This gap is particularly evident in arid coastal zones, where vegetation activity is highly sensitive by atmospheric conditions and short-term moisture variability rather than by precipitation alone (Holmgren et al., 2006; Vicente-Serrano et al., 2013; Green et al., 2017; Naik et al., 2025).

The Pacific coast of northern Peru provides a clear example of such a vulnerable acrid coastal system. Under normal conditions, the region is characterized by persistent aridity associated with the cold Humboldt Current, strong low-level inversions, and limited onshore moisture transport (Houston and Hartley, 2003; Garreaud et al., 2009). As a result, vegetation cover is sparse and highly dependent on episodic moisture inputs. During coastal El Niño events, however, nearshore sea surface temperatures increase significantly, and low-level circulation weakens or reverses. These changes allow moist air from the equatorial Pacific to penetrate inland, leading to a sharp enhancement of atmospheric moisture supply over the coastal zone (Garreaud, 2018; Takahashi et al., 2018; Rodriguez-Morata et al., 2019). These events represent natural experiments in which atmospheric moisture supply to an otherwise water-limited ecosystem is abruptly increased, providing an opportunity to examine how vegetation responds to transient moisture anomalies (Zhao et al., 2025).

The 2017 coastal El Niño in northern Peru was one of the strongest events observed along the eastern Pacific, producing pronounced anomalies in regional circulation and atmospheric moisture transport (Garreaud, 2018; Takahashi et al., 2018; Rocha et al., 2024). Most previous studies have primarily focused on this event from a meteorological and hydrological aspects, with emphasis on extreme rainfall, flooding, and associated socio-economic impacts (Rodriguez-Morata et al., 2019; Hong et al., 2024). However, the same circulation anomalies that produced heavy precipitation also modified the regional atmospheric moisture environment. These changes affected moisture transport pathways and near-surface humidity conditions over coastal land areas (Garreaud, 2018).The extent to which such moisture transport anomalies were reflected in terrestrial vegetation dynamics, however, has received little attention. Evidence linking the 2017 coastal El Niño to detectable vegetation responses remains limited (Sedighkia et al., 2024).

In this study, we investigate the relationship between atmospheric moisture transport anomalies and vegetation response in arid coastal ecosystems, using the 2017 coastal El Niño in northern Peru as a case study. We first characterize anomalous moisture transport pathways associated with the event through backward trajectory analysis, with attention to changes in moisture source regions and transport intensity. We then assess the statistical relationship between ocean-to-land water vapor transport (WVT) and vegetation activity, represented by the leaf area index (LAI), across coastal buffer zones and broader land areas. Short-lived atmospheric anomalies have been shown to induce measurable vegetation responses and, in some cases, to produce legacy effects on ecosystem productivity (Bastos et al., 2020; Zhi et al., 2024). This study focuses on whether transient anomalies in atmospheric moisture supply reflected in vegetation activity under extreme climate variability. The results offer process-based insight relevant to ecological restoration in water-limited coastal environments.

## 2 Data and methods

### 2.1 Study region and datasets

This study focuses on arid coastal ecosystems along the northern Peruvian coast, with particular attention to the Piura region (4°38′S, 79°43′W). Vegetation cover is sparse in this area. It is sensitive to variability in atmospheric moisture conditions. The regional climate is influenced by strong ocean-atmosphere coupling, persistent low-level inversions under normal conditions, and anomalous moisture intrusions during coastal El Niño events (Garreaud, 2018; Takahashi et al., 2018). These feathers make the region suitable for examining the relationship between atmospheric moisture supply and terrestrial vegetation activity.

Two primary categories of datasets were used. The first consists of meteorological field used to drive backward trajectory simulations and to quantify ocean-to-land water moisture transport. The second consists of reanalysis-based vegetation data to characterize terrestrial vegetation activity. Backward trajectories were computed with HYSPLIT (Stein et al., 2015). The model was driven by NOAA’s Global Data Assimilation System one-degree archive (GDAS1), which provides global three-dimensional meteorological variables on a 1° latitude-longitude grid with 3-hourly temporal resolution and standard pressure levels (Laboratory, 2004). This dataset is distributed in HYSPLIT-compatible format for trajectory applications (Laboratory, 2004).

Vegetation activity was represented by the leaf area index (LAI). LAI was obtained from ERA5 monthly averaged data on single levels provided through the Conpernicus Climate Data Store (CDS) (Hersbach, 2023). Water vapor transport (WVT) used in the correlation analysis was derived from ERA5 atmospheric fields. Specifically, ERA5 hourly reanalysis data on pressure levels were used, including specific humidity (*q*), zonal wind (*u*), and meridional wind (*v*) at 18 pressure levels ranging from 1000 hPa to 150 hPa. The data have a horizontal resolution of 0.25° × 0.25° and were retrieved from the CDS (Hersbach, 2023). Monthly means were computed from the hourly fields (00, 06, 12, and 18 UTC) prior to the calculation of vertically integrated moisture transport. The specific computation of WVT is described in Section 2.3. All datasets were processed using consistent spatial and temporal settings to support comparison between atmospheric moisture variability and vegetation variability.

### 2.2 Characterization of atmospheric moisture transport

#### 2.2.1 HYSPLIT backward trajectory analysis

Atmospheric moisture transport from oceanic source regions toward the northern Peruvian coast was diagnosed using the Hybrid Single-Particle Lagrangian Integrated Trajectory (HYSPLIT) model developed by the NOAA Air Resources Laboratory. HYSPLIT computes air-parcel trajectories by integrating three-dimensional wind fields backward in time, enabling the identification of dominant transport pathways and moisture source regions associated with large-scale circulation variability.

Seventy-two-hour backward trajectories were calculated to represent low-level atmospheric inflow relevant to near-surface moisture availability over land. To justify the selection of 2009 as the reference year and to distinguish background conditions from the 2017 coastal El Niño event, we examined sea surface temperature (SST) anomalies in the Niño1 + 2 region (0-10°S, 80-90°W) relative to the 1981–2010 climatology. March 2017 exhibited pronounced positive SST anomalies in Niño1 + 2 (Mar anomaly = +1.68 °C; JFM = +1.36 °C; FMA = +1.41 °C), consistent with the documented coastal El Niño. In contrast, March 2009 showed negative Niño1 + 2 anomalies (Mar anomaly = -0.75 °C; JFM = -0.50 °C; FMA = -0.29 °C), indicating the absence of coastal warming and representing a non-coastal El Niño baseline year. Basin-scale background conditions were also evaluated using the Oceanic Niño Index (ONI), which indicated weak La Niña conditions during early 2009. Trajectories were released every three hours (eight trajectories per day) throughout March for both a non-coastal El Niño reference year (2009) and the coastal El Niño year (2017), resulted in a total of 248 trajectories per month. All trajectories were initialized at Piura (4°38′S, 79°43′W) at a height of 100 m above ground level (AGL), corresponding to the atmospheric layer most directly influencing surface humidity conditions experienced by vegetation in arid coastal environments.

**Figure 1:**
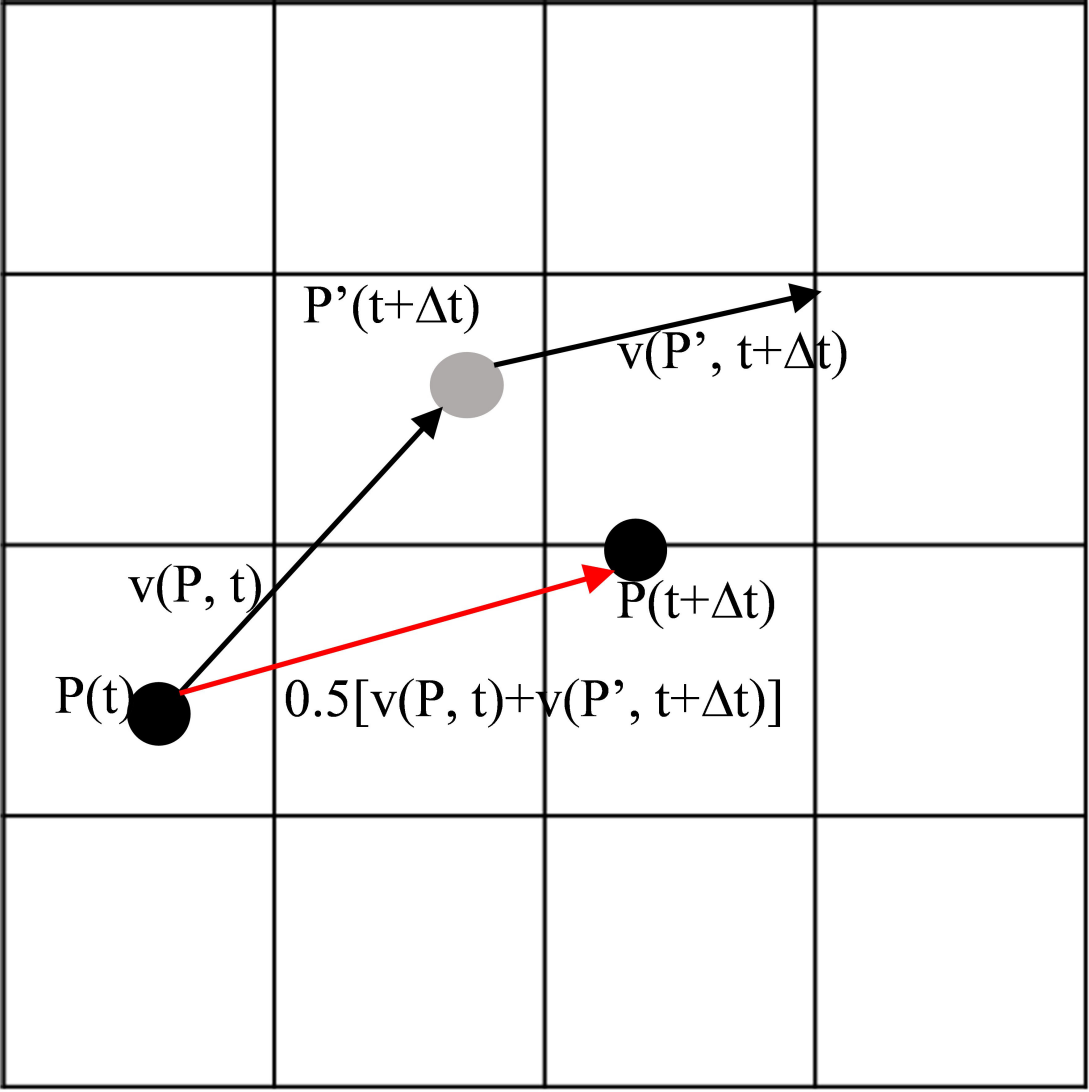
HYSPLIT trajectory integration schematic illustrating the trapezoidal method used to compute air-parcel positions from velocity fields.

Trajectory positions were computed using the trapezoidal (improved Euler) integration scheme implemented in HYSPLIT, which averages velocity vectors at successive time steps to reduce numerical error. The governing formulations used in this study are summarized in Equations (1–4). The parcel displacement can be expressed as:

(1) $$P(t+\Delta t)=P(t)+\frac{1}{2}[v(P,t)+v(P^{\prime },t+\Delta t)]\Delta t$$

where *P*(*t*) and P(*t*+Δ*t*) denote the parcel positions at time *t* and *t*+Δ*t*. The term *v*(*P*,*t*) represents the velocity vector at the parcel’s location and time. The conceptual framework of particle integration is illustrated in **Figure 1**.

#### 2.2.2 Trajectory clustering analysis

Because a large number of trajectories were generated (248), a clustering procedure was applied to objectively identify dominant transport pathways and to reduce redundancy among individual trajectories. Clustering was performed using the total spatial variance (TSV) criterion implemented in HYSPLIT, which groups trajectories based on their spatial similarity.

For a given cluster, TSV is defined as

(2) $$\text{TSV}=\sum_{i=1}^{N}[{\left(x_{i}-\bar{x}\right)}^{2}+{\left(y_{i}-\bar{y}\right)}^{2}+{\left(z_{i}-\bar{z}\right)}^{2}]$$

where *x_i_*, *y_i_*, *z_i_* denote the coordinates of the *i*-th trajectory and $\bar{x},\bar{y},\bar{z}$ represent the mean position of the cluster. The clustering algorithm iteratively merges trajectories in a way that minimizes the increase in TSV, thereby preserving physically coherent transport patterns.

The optimal number of clusters was determined by examining the percentage change in TSV between successive clustering steps. A sharp increase in TSV was interpreted as an indication that further merging would combine distinct flow regimes. The resulting clusters were evaluated for physical consistency in terms of trajectory direction, altitude range, and source region, ensuring that each cluster represents a meaningful atmospheric transport pathway relevant to moisture supply. These analyses are used to characterize variability in atmospheric moisture supply relevant to terrestrial vegetation conditions.

### 2.3 Vegetation activity and LAI-WVT analysis

#### 2.3.1 Leaf area index data

Vegetation activity was characterized using leaf area index (LAI), defined as the total one-sided leaf area per unit ground area. LAI integrates information on vegetation density, canopy structure, and photosynthetic potential and is widely used as an indicator of ecosystem state and vegetation dynamics in climate-ecosystem studies. Monthly LAI data were obtained from remotely sensed products covering the study period. The data were spatially aggregated to match the resolution of the atmospheric moisture diagnostics. To focus on coastal ecosystems most directly influenced by ocean-to-land moisture transport, LAI was analyzed within coastal buffer zones extending a few grid cells inland from the global coastline, as well as over global land areas.

To focus on coastal ecosystems most directly influenced by ocean-to-land moisture transport, LAI was analyzed within coastal buffer zones, defined as land grid cells extending two grid cells inland from the global coastline. Given the 0.25° spatial resolution of the dataset, this corresponds to an inland distance of approximately 0.5°. The same correlation analysis was also applied to all global land grid cells to characterize vegetation–moisture relationships at broader spatial scales.

#### 2.3.2 Water vapor transport metrics

Atmospheric moisture supply was quantified using water vapor transport (WVT), derived from vertically integrated moisture fluxes. The vertically integrated moisture flux vector Q is defined as

(3) $$\text{Q}=\frac{1}{g}\int_{p_{t}}^{p_{s}}qVdp$$

where *q* is specific humidity, **V**=(*u*,*v*) is the horizontal wind vector, *p_s_* and *p_t_* denote surface pressure and the upper integration limit, respectively, and *g* is gravitational acceleration.

To characterize ocean-to-land moisture exchange, WVT was evaluated in an aggregated diagnostic form representative of moisture inflow toward coastal regions. Spatially averaged WVT values were calculated for coastal interface zones, providing a measure of variability in atmospheric moisture supply associated with large-scale circulation anomalies. In this study, WVT is used as an indicator of atmospheric moisture availability rather than as a closed moisture budget component.

#### 2.3.3 Statistical analysis of LAI-WVT relationships

The relationship between vegetation activity and atmospheric moisture supply was examined using statistical association analysis between LAI and WVT. Pearson correlation coefficients were calculated to quantify the strength and direction of contemporaneous associations:

(4) $$r=\frac{\sum_{i=1}^{n}({\text{WVT}}_{i}-\bar{\text{WVT}})({\text{LAI}}_{i}-\bar{\text{LAI}})}{\sqrt{\sum_{i=1}^{n}{\left({\text{WVT}}_{i}-\bar{\text{WVT}}\right)}^{2}}\sqrt{\sum_{i=1}^{n}{\left({\text{LAI}}_{i}-\bar{\text{LAI}}\right)}^{2}}}$$

where WVT*_i_* and LAI*_i_* denote temporally collocated monthly values of water vapor transport and leaf area index at each grid point, and the overbars indicate temporal means over the analysis period. The sample size *n* corresponds to the number of monthly observations used in the correlation analysis. Correlation analyses were conducted across coastal buffer zones and broader land regions to identify spatial patterns in vegetation-moisture coupling.

The analysis focuses on contemporaneous relationships and is intended to provide a first-order diagnostic of how variability in atmospheric moisture transport coincides with vegetation activity under contrasting climate conditions. The results are interpreted in a descriptive sense rather than as evidence of direct causality.

## 3 Results

### 3.1 Anomalous atmospheric moisture transport during the 2017 coastal El Niño

#### 3.1.1 Backward trajectory comparison between 2009 and 2017

**Figure 2:**
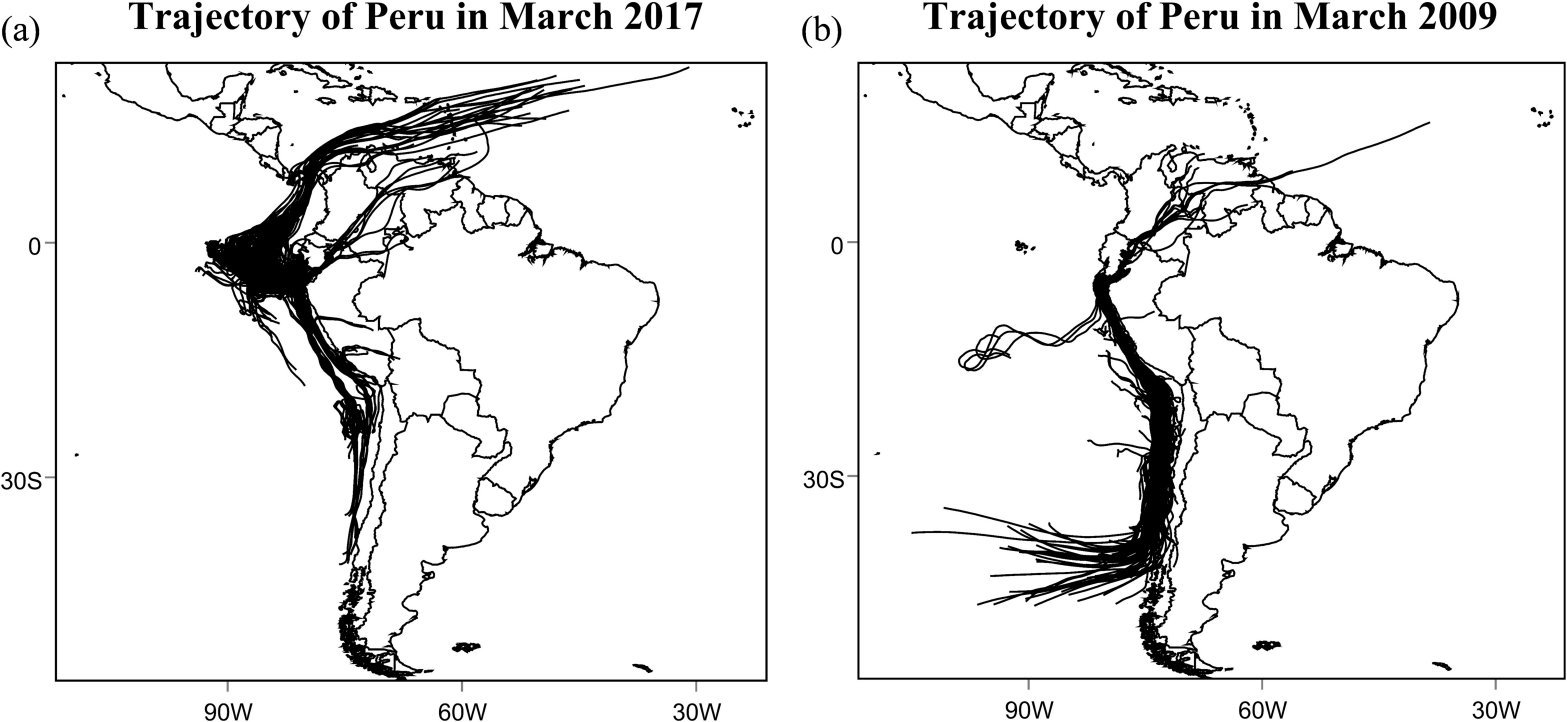
Backward trajectories of air parcels arriving at Piura in March 2017 (left) and March 2009 (right).

Backward trajectories computed using the HYSPLIT model reveal pronounced contrasts in atmospheric moisture transport pathways between the climatologically normal year 2009 and the coastal El Niño year 2017. **Figure 2** compares the 72-h backward trajectories arriving at Piura during March of the two years.

In March 2009, air parcels reaching the northern Peruvian coast predominantly originated from the southeastern Pacific Ocean and followed coherent, near-shore pathways aligned with the climatological southeast trade winds. These trajectories were largely confined to the lower troposphere, typically below the 850-hPa level, and remained closely parallel to the coastline. Such transport pathways are characteristic of normal conditions along the Peruvian coast, where cold upwelling associated with the Humboldt Current and a strong low-level inversion limit inland penetration of moist air.

In contrast, trajectories during March 2017 exhibit a significantly different structure. A substantial fraction of air parcels originated from the equatorial Pacific, extending westward beyond 100° W before turning toward the South American coast. In addition to this dominant equatorial pathway, a secondary branch of trajectories traced back to inland tropical South America, indicating cross-continental transport from the Atlantic sector. The overall pattern reflects a reorganization of low-level circulation during the coastal El Niño, with enhanced zonal and meridional connectivity linking multiple moisture source regions to the northern Peruvian coast.

These differences highlight a fundamental shift in atmospheric moisture supply between the two years. While 2009 was characterized by a relatively uniform and shallow marine inflow, the 2017 event featured more diverse and spatially extensive transport pathways capable of delivering moist air from both oceanic and continental sources.

#### 3.1.2 Cluster characteristics and dominant transport pathways

**Figure 3:**
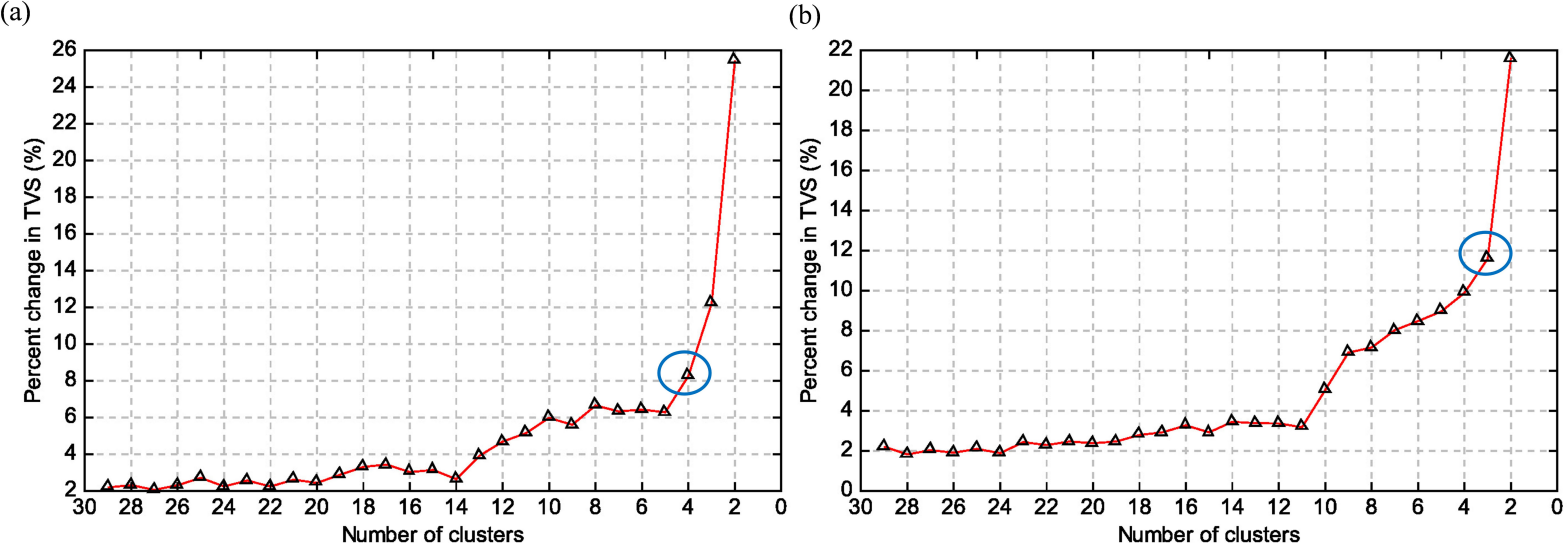
Percent change in total spatial variance (TVS) by cluster number for March 2017 (left) and March 2009 (right). Circles show the chosen cluster counts.

To objectively characterize the dominant moisture transport regimes, the backward trajectories were grouped using a total spatial variance (TSV) clustering approach. The percentage change in TSV as a function of cluster number is shown in **Figure 3** and was used to identify the optimal number of representative clusters for each year.

**Figure 4:**
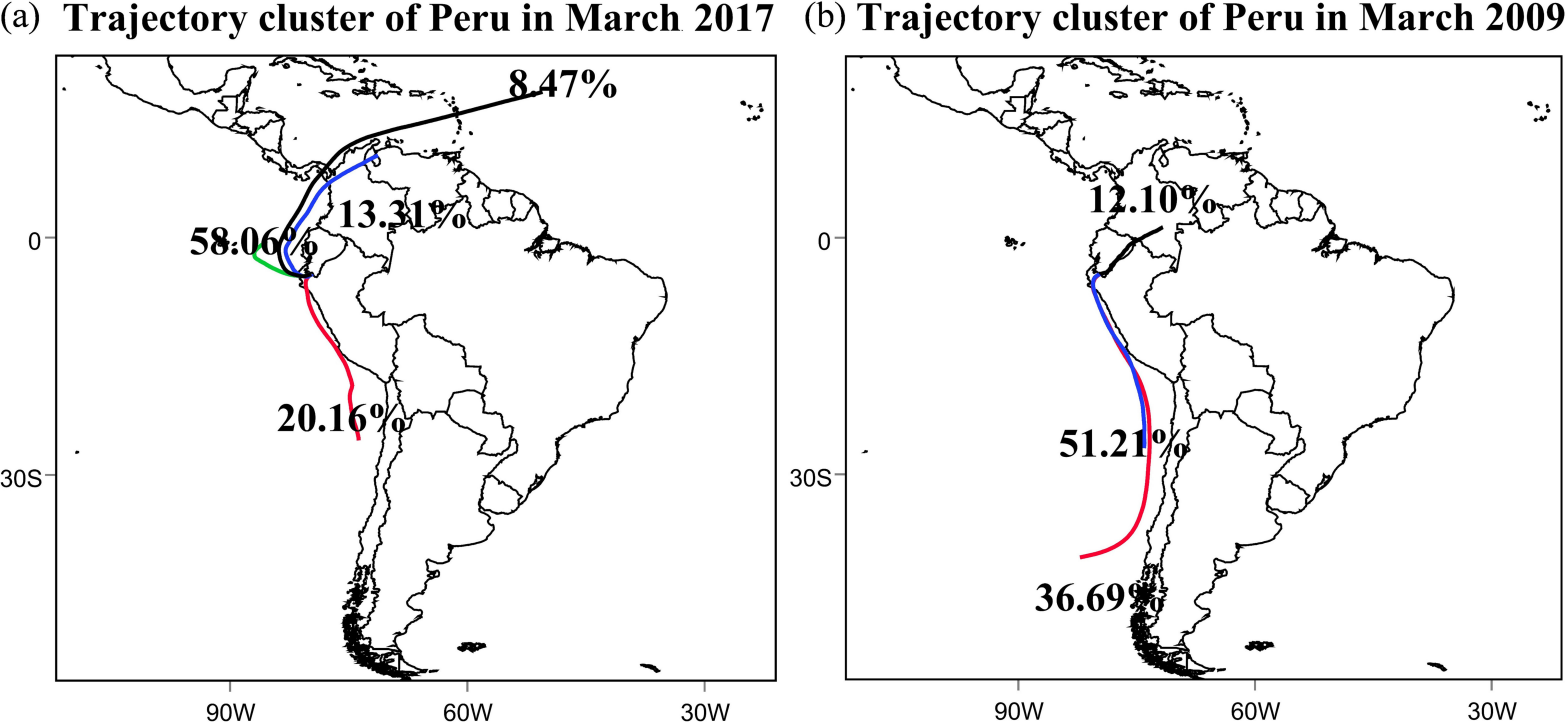
Clustered backward trajectories for air parcels arriving at Piura in March 2017 (left) and March 2009 (right). The colors show different trajectory clusters.

For March 2009, three major clusters were identified. Combined with the results in **Figure 4**, two of these clusters accounted for nearly 90% of all trajectories and represented transport from the southeastern Pacific along the coast. These clusters exhibited similar spatial characteristics and reflected the persistence of climatological trade-wind-driven flow. A third, less frequent cluster originated from inland South America, indicating limited continental moisture recycling under normal conditions.

In contrast, four dominant clusters were identified for March 2017 (**Figure 4**), reflecting increased diversity in moisture source regions during the coastal El Niño. The largest cluster originated from the near-equatorial Pacific and accounted for approximately 60% of the total trajectories, highlighting the dominant role of equatorial oceanic inflow during the event. A second cluster followed the southeastern Pacific pathway but with altered orientation and frequency relative to 2009, consistent with a weakening of the climatological coastal flow. Two additional clusters traced air parcels from inland northern South America and, more distantly, from the tropical Atlantic, indicating enhanced cross-continental connectivity.

The emergence of these additional clusters in 2017 reflects the reorganization of large-scale circulation associated with the coastal El Niño. Rather than a single dominant marine source, atmospheric moisture reaching the northern Peruvian coast during the event was supplied by a combination of equatorial Pacific, coastal Pacific, and continental pathways.

#### 3.1.3 Humidity contrasts along transport pathways

**Figure 5:**
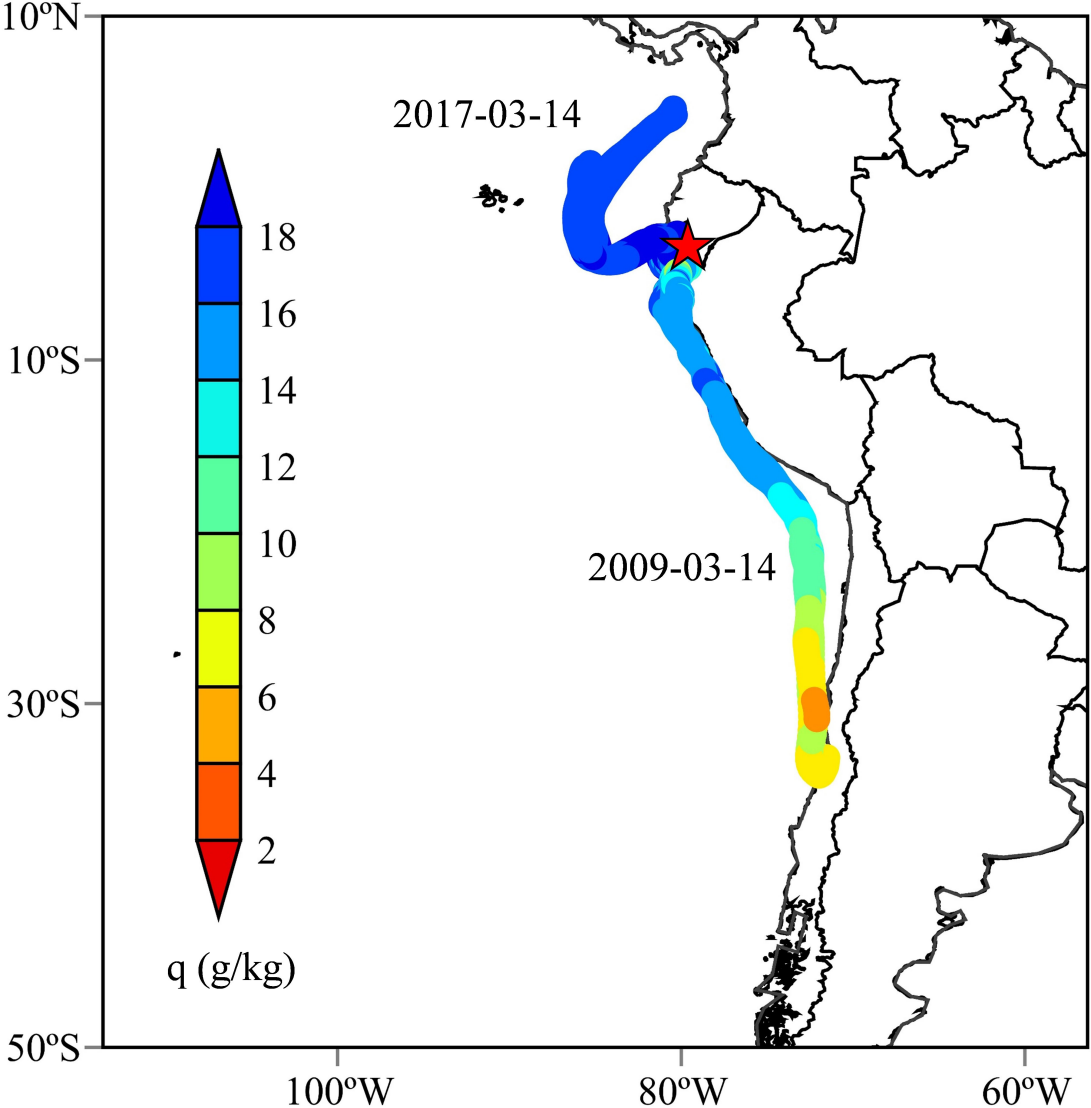
Backward trajectories and specific humidity (*q*, g/kg) on 14 March 2017 and 2009.

Specific humidity extracted along the backward trajectories provides further insight into the moisture characteristics of the identified transport regimes. **Figure 5** compares the humidity evolution of air parcels during representative periods in March 2009 and March 2017.

Under normal conditions in 2009, air parcels arriving at Piura maintained relatively low specific humidity values, generally below 10 g/kg throughout their transport history. Humidity exhibited limited variation along the trajectories, consistent with transport over cool ocean surfaces and weak evaporation under the influence of the Humboldt Current.

In contrast, trajectories during the 2017 coastal El Niño carried substantially higher moisture content. Specific humidity values frequently exceeded 15 g/kg, with a clear increase as air parcels approached the coast. This enhancement reflects both elevated sea surface temperatures in the eastern equatorial Pacific and intensified low-level convergence near the coastline. The presence of moist continental trajectories further contributed to maintaining high background humidity levels during the event.

The humidity contrasts between the two years demonstrate that the 2017 coastal El Niño was associated not only with altered transport pathways but also with a pronounced increase in atmospheric moisture supply to the northern Peruvian coast. These combined dynamical and thermodynamic changes resulted in a moisture environment that was clearly distinct from normal conditions.

### 3.2 Vegetation-moisture relationships inferred from LAI-WVT analysis

#### 3.2.1 LAI-WVT correlations within coastal buffer zones

**Figure 6:**
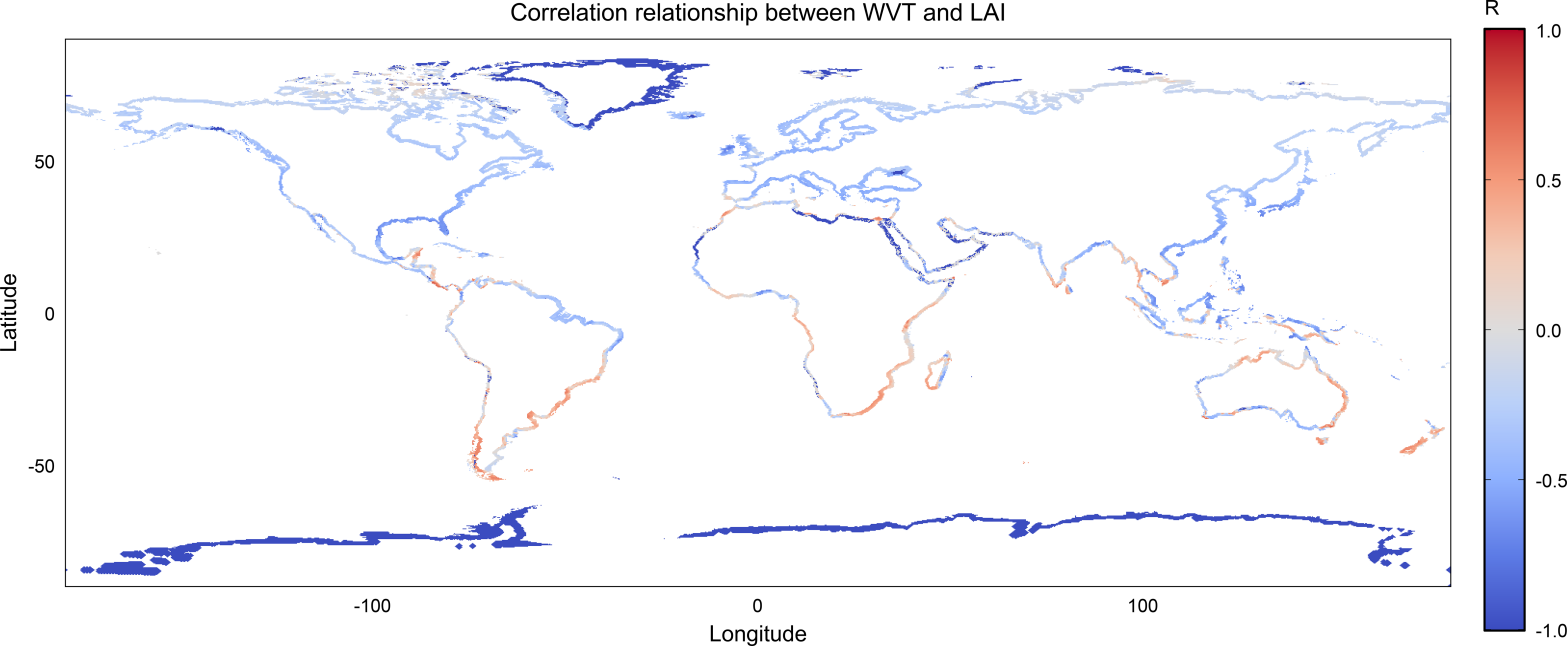
Spatial distribution of Pearson correlations between LAI and coastal ocean-to-land water vapor transport (WVT), calculated for land grid cells within a two-grid-cell buffer zone inland from the global coastline.

**Figure 6** shows the spatial distribution of Pearson correlations between LAI and coastal WVT within a two-grid-cell buffer zone inland from the global coastline. The correlation pattern is highly heterogeneous along the global coastline, with adjacent coastal segments often exhibiting contrasting correlation signs.

Along the eastern and western coast of South America, positive LAI-WVT correlations are visible over extended coastal stretches, particularly in the subtropical and mid-latitude portions of the continent. These positive correlations indicate that, within these coastal buffer zones, enhanced ocean-to-land moisture transport tends to coincide with higher vegetation activity. Similar positive relationships are also observed along parts of the southern African coastline and portions of northern and eastern Australia.

In contrast, large sections of the tropical coastal belt display predominantly negative LAI-WVT correlations. This pattern is evident along the coasts of South and parts Southeast Asia, parts of West Africa, and sections of Central America. In these regions, increased coastal WVT is associated with reduced or suppressed LAI, suggesting that vegetation activity does not respond positively to enhanced atmospheric moisture transport at the coastal interface.

High-latitude coastal regions in both hemispheres are characterized mainly by negative correlations, with particularly consistent negative values along polar and subpolar coastlines. These areas exhibit relatively uniform correlation patterns compared with the more fragmented structure seen in the subtropics and tropics.

Overall, the coastal buffer analysis reveals that the relationship between atmospheric moisture transport and vegetation activity varies strongly with latitude and regional climate setting. Positive LAI-WVT correlations tend to occur in coastal environments where vegetation is likely moisture-limited, whereas negative correlations dominate in humid tropical and high-latitude coastal regions.

#### 3.2.2 LAI-WVT correlations across global land areas

**Figure 7:**
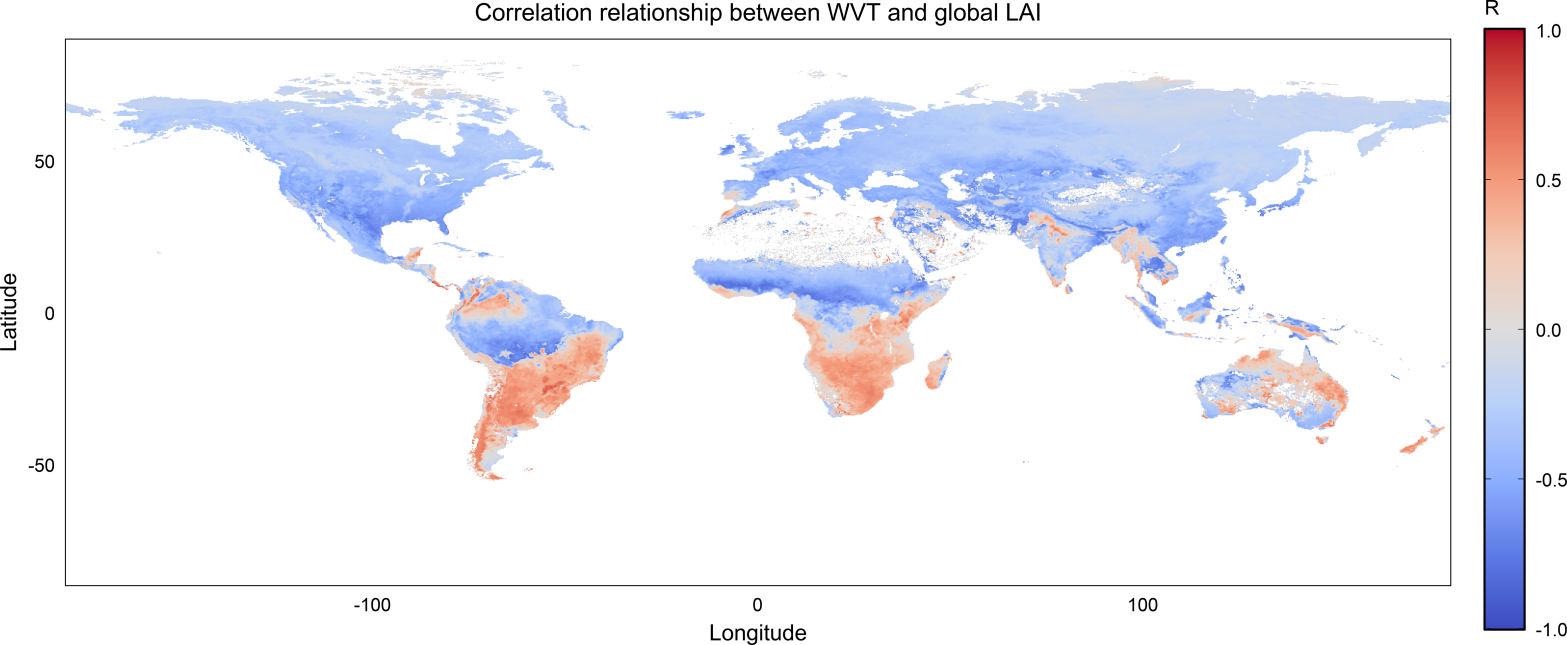
Same as **Figure 6**, but for all global land grid cells.

**Figure 7** extends the analysis to all global land pixels, providing a broader view of vegetation-moisture coupling beyond the immediate coastal zone. At the global scale, negative LAI-WVT correlations dominate much of the Northern Hemisphere, particularly across North America, Europe, and large areas of northern and central Eurasia. These regions form extensive, spatially coherent zones where increased atmospheric moisture transport does not correspond to enhanced vegetation activity.

In contrast, large contiguous areas of positive LAI-WVT correlation are evident across the Southern Hemisphere. Pronounced positive correlations are observed over southern South America, southern Africa, and northern and eastern Australia. These regions largely coincide with areas where vegetation growth is sensitive to moisture availability, and where variations in atmospheric moisture transport appear to be more closely linked to vegetation dynamics.

Tropical land regions exhibit a more complex and spatially fragmented pattern. Portions of the Amazon Basin and equatorial Africa show mixed correlation signs, with positive and negative values occurring in close proximity. This spatial variability suggests that, in humid tropical environments, vegetation responses to atmospheric moisture transport are modulated by additional factors beyond large-scale moisture supply. In the Amazon Basin, the predominance of negative LAI-WVT correlations suggests that vegetation activity is not primarily moisture-limited. In this humid tropical environment, enhanced ocean-to-land moisture transport is often associated with increased cloudiness and convective activity, which may reduce incoming solar radiation and constrain photosynthetic efficiency. As a result, stronger moisture transport does not necessarily translate into increased vegetation activity.

Compared with the coastal buffer results, the global land analysis highlights a clearer hemispheric contrast. Positive LAI-WVT correlations are more prevalent across Southern Hemisphere land areas, whereas negative correlations dominate across much of the Northern Hemisphere. This large-scale contrast indicates that the sensitivity of vegetation activity to atmospheric moisture transport depends strongly on background climatic conditions and prevailing ecological constraints.

In polar regions, the difference between **Figures 6**, **7** reflects the contrasting spatial sampling domains. Within the coastal buffer zone (**Figure 6**), correlations are computed only for narrow coastal land margins, where surface-atmosphere exchange remains dynamically connected to adjacent oceanic moisture transport. These areas exhibit relatively coherent negative correlations. However, when all land pixels are included (**Figure 7**), extensive inland ice-covered regions with near-zero LAI variability are incorporated into the analysis. In these interior polar areas, vegetation activity is primarily limited by temperature rather than moisture availability, and the statistical relationship with WVT becomes weak or spatially inconsistent. As a result, the broader global-land analysis reduces the apparent coherence observed along the coastal margins.

## 4 Discussion

This study analyzes atmospheric moisture transport during the 2017 coastal El Niño with a global-scale statistical assessment of vegetation-moisture relationships to examine how transient changes in atmospheric moisture supply may influence vegetation activity in arid coastal ecosystems. The results show a well-documented circulation anomaly over northern Peru with broader LAI-WVT patterns. This indicates that atmospheric moisture transport can constrain terrestrial vegetation activity.

The trajectory analysis for northern Peru shows that the 2017 coastal El Niño was characterized by a fundamental reorganization of low-level moisture transport pathways. Compared with climatologically normal conditions, moisture reaching the Peruvian coast during the event originated from more diverse and distant source regions, including the equatorial Pacific and continental South America, and carried substantially higher specific humidity. These changes reflect a weakening of the climatological coastal circulation and enhanced zonal connectivity, which together produced an anomalously moist near-surface atmospheric environment. While previous studies have emphasized the hydrological impacts of the 2017 event, the present results highlight that the same circulation anomalies also altered the atmospheric moisture conditions experienced by terrestrial ecosystems along the coast. This is consistent with earlier work highlighting the sensitivity of vegetation activity to atmospheric moisture availability in water-limited environments (Nemani et al., 2003; Seneviratne et al., 2010; Yan et al., 2025).

The global LAI-WVT analysis places this case study in a broader ecological context. Correlation patterns observed at both coastal and continental scales indicate that vegetation responses to atmospheric moisture transport depend strongly on background climatic conditions. Positive LAI-WVT relationships are most evident in areas where vegetation growth is likely limited by water availability, particularly across large parts of the Southern Hemisphere and along selected arid and semi-arid coastlines. In contrast, negative or weak correlations dominate in humid tropical regions and across much of the Northern Hemisphere, where vegetation activity is influenced by additional constraints such as radiation, temperature, or seasonal phenology.

The predominantly negative LAI-WVT relationships over large portions of the Amazon further illustrate this contrast between moisture- and energy-limited ecosystems. Unlike arid regions where additional moisture directly alleviates water stress, the Amazon rainforest typically operates under conditions of abundant baseline moisture. In such environments, enhanced atmospheric moisture transport is frequently associated with increased cloud cover and reduced solar radiation, potentially limiting photosynthetic activity despite sufficient water availability. As a result, vegetation dynamics in the Amazon may respond more strongly to variations in radiation and atmospheric stability than to additional moisture supply. These patterns suggest that atmospheric moisture transport does not operate in isolation but instead interacts with background climatic conditions to shape ecosystem responses.

The apparent differences between the coastal-buffer and full land-domain analyses over Greenland and Antarctica further illustrate this spatial dependence. Within the narrow coastal margins (**Figure 6**), correlations are calculated only for land grid cells directly adjacent to the ocean, where surface-atmosphere exchanges remain dynamically connected to moisture transport variability. In these polar coastal zones, coherent negative correlations emerge. However, when the analysis includes all land pixels (**Figure 7**), extensive inland ice-covered regions characterized by near-zero LAI variability are incorporated. In these interior areas, vegetation activity is primarily limited by temperature and radiation rather than atmospheric moisture supply, weakening the statistical association with WVT. This contrast indicates that vegetation responses in polar interiors are primarily temperature-limited, whereas coastal margins remain more directly influenced by atmosphere-ocean interactions. Such region-dependent controls are consistent with previous studies showing that water, energy, and phenological constraints jointly regulate vegetation dynamics across different climate regimes (Nemani et al., 2003; Seddon et al., 2016).

Overall, the Peru case and the global correlation patterns point to atmospheric moisture transport as an often overlooked component of vegetation-climate coupling. In moisture-limited environments, variability in ocean-to-land moisture transport may modulate near-surface humidity, precipitation efficiency, and evaporative demand, thereby influencing vegetation activity even in the absence of sustained rainfall anomalies. Extreme climate events such as coastal El Niño events can temporarily relax atmospheric moisture constraints in arid coastal systems, creating short-lived conditions that may support vegetation growth or recovery. From an ecological restoration perspective, such transient moisture anomalies may represent critical windows during which degraded vegetation systems experience reduced water stress, although the persistence and ecological significance of these effects remain uncertain.

Beyond the scientific findings, combining atmospheric diagnostics with vegetation indicators involved several practical challenges. First, moisture transport metrics represent large-scale circulation variability projected onto coastal boundaries, whereas LAI reflects spatially averaged vegetation conditions at the grid-cell scale. This spatial mismatch complicates direct physical interpretation. Second, ocean-to-land moisture transport does not directly measure precipitation or soil moisture availability, but instead describes atmospheric moisture supply potential. As a result, the linkage between WVT and vegetation activity is indirect and may be mediated by local hydrological processes. Finally, integrating an event-scale circulation analysis with global statistical correlations required careful separation of dynamical interpretation from broader climatological associations.

Several limitations of the present analysis should be acknowledged. The LAI-WVT relationships examined here are based on contemporaneous statistical associations and do not provide evidence for causal relationships between atmospheric moisture transport and vegetation response. The analysis does not explicitly account for lead-lag effects, local precipitation processes, soil moisture dynamics, or human land management, all of which can influence vegetation activity. Despite these limitations, the combined trajectory-based and statistical framework provides a useful starting point for integrating atmospheric moisture transport into assessments of vegetation-climate interactions.

Future work could extend this framework by incorporating lead-lag analyses to better resolve the timing of vegetation responses, coupling moisture transport diagnostics with soil moisture and precipitation data, or focusing on specific ecological regions to examine process-level mechanisms in greater detail. Such efforts would help clarify the extent to which atmospheric moisture transport influences vegetation resilience and recovery under increasing climate variability. Nevertheless, the results presented here underscore that large-scale atmospheric moisture transport is a relevant and spatially variable factor in shaping vegetation activity, particularly in arid coastal ecosystems where water availability remains a dominant constraint.

## 5 Conclusions

This study examined the relationship between atmospheric moisture transport and vegetation activity by combining a process-based analysis of the 2017 coastal El Niño in northern Peru with a global assessment of LAI-WVT statistical relationships. The results highlight that large-scale atmospheric moisture transport can substantially alter near-surface moisture conditions in arid coastal environments and that vegetation responses to such variability are highly dependent on regional climatic context.

The trajectory-based analysis for northern Peru shows that the 2017 coastal El Niño was associated with a pronounced reorganization of low-level moisture transport pathways, characterized by enhanced inflow from the equatorial Pacific and increased moisture content of air parcels reaching the coast. These circulation anomalies produced an atmospheric environment strongly different from climatologically normal conditions, emphasizing that extreme climate events can modify moisture availability beyond local precipitation alone.

At the global scale, LAI-WVT correlations reveal a heterogeneous pattern of vegetation-moisture coupling. Positive associations are most evident in moisture-limited regions, particularly across large areas of the Southern Hemisphere and selected arid and semi-arid coastal zones, whereas negative or weak correlations dominate in humid tropical regions and much of the Northern Hemisphere. These contrasting patterns indicate that the influence of atmospheric moisture transport on vegetation activity is neither uniform nor universal, but instead reflects underlying climatic constraints and ecosystem sensitivities.

The results suggest that atmospheric moisture transport represents an important but often underappreciated component of vegetation-climate interactions. In arid coastal ecosystems, transient enhancements in moisture transport, such as those associated with coastal El Niño events, may temporarily relax atmospheric moisture limitations and coincide with increased vegetation activity. The results suggest that variability in ocean-to-land moisture transport is closely associated with vegetation dynamics in specific environmental settings.

Overall, this study underscores the value of integrating atmospheric moisture diagnostics with vegetation observations to better understand ecosystem responses to climate variability. Future research incorporating lead-lag analyses, additional hydrological variables, and region-specific ecological assessments will be essential for clarifying the mechanisms through which atmospheric moisture transport influences vegetation resilience and recovery under a changing climate.

## Data Availability

GDAS1 meteorological data are provided by the NOAA Air Resources Laboratory and are available at https://www.ready.noaa.gov/gdas1.php. ERA5 reanalysis data are available from the European Centre for Medium-Range Weather Forecasts (ECMWF) via https://cds.climate.copernicus.eu/datasets/reanalysis-era5-pressure-levels?tab=overview and https://cds.climate.copernicus.eu/datasets/reanalysis-era5-single-levels-monthly-means?tab=download.

## References

[B1] Bastos A. Ciais P. Friedlingstein P. Sitch S. Pongratz J. Fan L. . (2020). Direct and seasonal legacy effects of the 2018 heat wave and drought on European ecosystem productivity. Sci. Adv. 6, eaba2724. doi: 10.1126/sciadv.aba2724, PMID: 32577519 PMC7286671

[B2] Cai W. Borlace S. Lengaigne M. Van Rensch P. Collins M. Vecchi G. . (2014). Increasing frequency of extreme El Niño events due to greenhouse warming. Nat. Clim. Change 4, 111–116. doi: 10.1038/nclimate2100, PMID: 41896565

[B3] Chaves M. M. Maroco J. P. Pereira J. S. (2003). Understanding plant responses to drought—from genes to the whole plant. Funct. Plant Biol. 30, 239–264. doi: 10.1071/FP02076, PMID: 32689007

[B4] D’Odorico P. Bhattachan A. Davis K. F. Ravi S. Runyan C. W. (2013). Global desertification: Drivers and feedbacks. Adv. Water Resour. 51, 326–344. doi: 10.1016/j.advwatres.2012.01.013, PMID: 41909469

[B5] Garreaud R. D. (2018). A plausible atmospheric trigger for the 2017 coastal El Niño. Int. J. Climatol. 38, e1296-e1302. doi: 10.1002/joc.5426. PMID: 41784088

[B6] Garreaud R. D. Vuille M. Compagnucci R. Marengo J. (2009). Present-day south American climate. Palaeogeogr. Palaeoclimatol. Palaeoecol. 281, 180–195. doi: 10.1016/j.palaeo.2007.10.032, PMID: 41909469

[B7] Gimeno L. Stohl A. Trigo R. M. Dominguez F. Yoshimura K. Yu L. . (2012). Oceanic and terrestrial sources of continental precipitation. Rev. Geophys. 50. doi: 10.1029/2012RG000389. PMID: 40890438

[B8] Green J. K. Konings A. G. Alemohammad S. H. Berry J. Entekhabi D. Kolassa J. . (2017). Regionally strong feedbacks between the atmosphere and terrestrial biosphere. Nat. Geosci. 10, 410–414. doi: 10.1038/ngeo2957, PMID: 31709007 PMC6839712

[B9] Hersbach H. (2023). ERA5 monthly averaged data on single levels from 1940 to present. https://cds.climate.copernicus.eu/datasets/reanalysis-era5-single-levels-monthly-means

[B10] Holmgren M. Scheffer M. Ezcurra E. Gutiérrez J. R. Mohren G. M. (2001). El Niño effects on the dynamics of terrestrial ecosystems. Trends Ecol. Evol. 16, 89–94. doi: 10.1016/S0169-5347(00)02052-8, PMID: 11165707

[B11] Holmgren M. Stapp P. Dickman C. R. Gracia C. Graham S. Gutiérrez J. R. . (2006). Extreme climatic events shape arid and semiarid ecosystems. Front. Ecol. Environ. 4, 87–95. doi: 10.1890/1540-9295(2006)004[0087:ECESAA]2.0.CO;2

[B12] Holton J. R. Dmowska R. (1989). El Niño, La Niña, and the southern oscillation (San Diego, CA, USA: Academic Press).

[B13] Hong L. Zhang Z. Huang G. (2024). Characterization of flood risk in Japan in times of climate change and aging society. J. Environ. Inf. 44, 100–111. doi: 10.3808/jei.202400525

[B14] Houston J. Hartley A. J. (2003). The central Andean west‐slope rainshadow and its potential contribution to the origin of hyper‐aridity in the Atacama Desert. Int. J. Climatology: A J. R. Meteorological Soc. 23, 1453–1464. doi: 10.1002/joc.938, PMID: 41889077

[B15] Maestre F. T. Quero J. L. Gotelli N. J. Escudero A. Ochoa V. Delgado-Baquerizo M. . (2012). Plant species richness and ecosystem multifunctionality in global drylands. Science 335, 214–218. doi: 10.1126/science.1215442, PMID: 22246775 PMC3558739

[B16] Martinez J. A. Dominguez F. (2014). Sources of atmospheric moisture for the La Plata River basin. J. Clim. 27, 6737–6753. doi: 10.1175/JCLI-D-14-00022.1, PMID: 40797873

[B17] McPhaden M. J. Zebiak S. E. Glantz M. H. (2006). ENSO as an integrating concept in earth science. Science 314, 1740–1745. doi: 10.1126/science.1132588, PMID: 17170296

[B18] Naik S. Begum M. Pradhan U. Panda U. Dash S. Mishra P. . (2025). The relationship between N/P/Si stoichiometry and phytoplankton blooms in the surf zone coastal waters, Southwestern Bay of Bengal. J. Environ. Inf. 45, 118–128. doi: 10.3808/jei.202500537

[B19] Nemani R. R. Keeling C. D. Hashimoto H. Jolly W. M. Piper S. C. Tucker C. J. . (2003). Climate-driven increases in global terrestrial net primary production from 1982 to 1999. Science 300, 1560–1563. doi: 10.1126/science.1082750, PMID: 12791990

[B20] Nichols C. R. Zinnert J. Young D. R. (2018). “ Degradation of coastal ecosystems: causes, impacts and mitigation efforts,” in Tomorrow's Coasts: Complex and Impermanent (Cham, Switzerland: Springer), 119–136.

[B21] NOAA Air Resources Laboratory (ARL) . (2004). Global Data Assimilation System (GDAS1) Archive Information. Available online at: https://www.ready.noaa.gov/gdas1.php.

[B22] Novick K. A. Ficklin D. L. Stoy P. C. Williams C. A. Bohrer G. Oishi A. C. . (2016). The increasing importance of atmospheric demand for ecosystem water and carbon fluxes. Nat. Clim. Change 6, 1023–1027. doi: 10.1038/nclimate3114, PMID: 41896565

[B23] Noy-Meir I. (1973). Desert ecosystems: environment and producers. Annu. Rev. Ecol. Systematics 4, 25–51. doi: 10.1146/annurev.es.04.110173.000325, PMID: 41139587

[B24] Reynolds J. F. Smith D. M. S. Lambin E. F. Turner B. Mortimore M. Batterbury S. P. . (2007). Global desertification: building a science for dryland development. Science 316, 847–851. doi: 10.1126/science.1131634, PMID: 17495163

[B25] Rocha V. Santos C. Silva R. (2024). Estimation of water surface reflectance and total suspended solid using MODIS images for a reservoir in the Brazilian semiarid region. J. Environ. Inf. 44, 112–125. doi: 10.3808/jei.202400527

[B26] Rodriguez-Morata C. Díaz H. Ballesteros-Canovas J. A. Rohrer M. Stoffel M. (2019). The anomalous 2017 coastal El Niño event in Peru. Clim. Dyn. 52, 5605–5622. doi: 10.1007/s00382-018-4466-y, PMID: 41913934

[B27] Seddon A. W. Macias-Fauria M. Long P. R. Benz D. Willis K. J. (2016). Sensitivity of global terrestrial ecosystems to climate variability. Nature 531, 229–232. doi: 10.1038/nature16986, PMID: 26886790

[B28] Sedighkia M. Datta B. Saeidipour P. (2024). An environmental operation of reservoirs through linking ecological storage model and evolutionary optimization. J. Environ. Inf. 43, 105–117. doi: 10.3808/jei.202400513

[B29] Seneviratne S. I. Corti T. Davin E. L. Hirschi M. Jaeger E. B. Lehner I. . (2010). Investigating soil moisture–climate interactions in a changing climate: A review. Earth Sci. Rev. 99, 125–161. doi: 10.1016/j.earscirev.2010.02.004, PMID: 41909469

[B30] Sivakumar M. V. Stefanski R. (2007). Climate and land degradation—an overview. Climate Land Degradation, 105–135. doi: 10.1007/978-3-540-72438-4, PMID: 41913934

[B31] Sodemann H. Schwierz C. Wernli H. (2008). Interannual variability of Greenland winter precipitation sources: Lagrangian moisture diagnostic and North Atlantic Oscillation influence. J. Geophysical Research: Atmospheres 113. doi: 10.1029/2007JD008503, PMID: 40890438

[B32] Stein A. F. Draxler R. R. Rolph G. D. Stunder B. J. Cohen M. D. Ngan F. (2015). NOAA’s HYSPLIT atmospheric transport and dispersion modeling system. Bull. Am. Meteorol. Soc 96, 2059–2077. doi: 10.1175/BAMS-D-14-00110.1, PMID: 40797873

[B33] Tahsin S. Medeiros S. C. Singh A. (2018). Assessing the resilience of coastal wetlands to extreme hydrologic events using vegetation indices: A review. Remote Sens. 10, 1390. doi: 10.3390/rs10091390, PMID: 41725453

[B34] Takahashi K. Aliaga-Nestares V. Avalos G. Bouchon M. Castro A. Cruzado L. . (2018). The 2017 coastal el nino. Bulletin of the American Meteorological Society. 99, S210–S242. doi: 10.1175/2018BAMSStateoftheClimate.1, PMID: 40797873

[B35] Trenberth K. E. Branstator G. W. Karoly D. Kumar A. Lau N. C. Ropelewski C. (1998). Progress during TOGA in understanding and modeling global teleconnections associated with tropical sea surface temperatures. J. Geophysical Research: Oceans 103, 14291–14324. doi: 10.1029/97JC01444, PMID: 40890438

[B36] Trenberth K. E. Fasullo J. T. Mackaro J. (2011). Atmospheric moisture transports from ocean to land and global energy flows in reanalyses. J. Clim. 24, 4907–4924. doi: 10.1175/2011JCLI4171.1, PMID: 40797873

[B37] Vicente-Serrano S. M. Gouveia C. Camarero J. J. Beguería S. Trigo R. López-Moreno J. I. . (2013). Response of vegetation to drought time-scales across global land biomes. Proc. Natl. Acad. Sci. 110, 52–57. doi: 10.1073/pnas.1207068110, PMID: 23248309 PMC3538253

[B38] Yan D. Qin T. Dong B. Lu J. Zhang X. Li C. . (2025). Mechanism of interaction between global terrestrial net emissions of carbon dioxide and water cycle. J. Environ. Inf. 45, 129–143. doi: 10.3808/jei.202500539

[B39] Zhao L. Jia K. Xia M. Yuan B. Tao G. Li J. . (2025). An improved habitat quality assessment model considering vegetation growth status: A case study of the Qinghai–Tibet Plateau. J. Environ. Inf. 46, 132–144. doi: 10.3808/jei.202500547

[B40] Zhi L. Xie X. Gao W. Li X. Bai J. Wang D. . (2024). Sediment grain size affects vegetation patterns in river-dominated deltas. J. Environ. Inf. 43, 118–128. doi: 10.3808/jei.202400514

